# Deciphering the Crosstalk Mechanisms of Wheat-Stem Rust Pathosystem: Genome-Scale Prediction Unravels Novel Host Targets

**DOI:** 10.3389/fpls.2022.895480

**Published:** 2022-06-21

**Authors:** Raghav Kataria, Rakesh Kaundal

**Affiliations:** ^1^Department of Plants, Soils, and Climate, College of Agriculture and Applied Sciences, Utah State University, Logan, UT, United States; ^2^Bioinformatics Facility, Center for Integrated BioSystems, Utah State University, Logan, UT, United States; ^3^Department of Computer Science, College of Science, Utah State University, Logan, UT, United States

**Keywords:** wheat, stem rust, computational modeling, effectors, interolog method, domain-based approach, disease resistance

## Abstract

*Triticum aestivum* (wheat), a major staple food grain, is affected by various biotic stresses. Among these, fungal diseases cause about 15–20% of yield loss, worldwide. In this study, we performed a comparative analysis of protein-protein interactions between two *Puccinia graminis* races (*Pgt* 21-0 and *Pgt* Ug99) that cause stem (black) rust in wheat. The available molecular techniques to study the host-pathogen interaction mechanisms are expensive and labor-intensive. We implemented two computational approaches (interolog and domain-based) for the prediction of PPIs and performed various functional analysis to determine the significant differences between the two pathogen races. The analysis revealed that *T. aestivum*-*Pgt* 21-0 and *T. aestivum*-*Pgt* Ug99 interactomes consisted of ∼90M and ∼56M putative PPIs, respectively. In the predicted PPIs, we identified 115 *Pgt* 21-0 and 34 *Pgt* Ug99 potential effectors that were highly involved in pathogen virulence and development. Functional enrichment analysis of the host proteins revealed significant GO terms and KEGG pathways such as *O*-methyltransferase activity (GO:0008171), regulation of signal transduction (GO:0009966), lignin metabolic process (GO:0009808), plastid envelope (GO:0009526), plant-pathogen interaction pathway (ko04626), and MAPK pathway (ko04016) that are actively involved in plant defense and immune signaling against the biotic stresses. Subcellular localization analysis anticipated the host plastid as a primary target for pathogen attack. The highly connected host hubs in the protein interaction network belonged to protein kinase domain including Ser/Thr protein kinase, MAPK, and cyclin-dependent kinase. We also identified 5,577 transcription factors in the interactions, associated with plant defense during biotic stress conditions. Additionally, novel host targets that are resistant to stem rust disease were also identified. The present study elucidates the functional differences between *Pgt* 21-0 and *Pgt* Ug99, thus providing the researchers with strain-specific information for further experimental validation of the interactions, and the development of durable, disease-resistant crop lines.

## Introduction

*Triticum aestivum* L. (family *Poaceae*) is one of the highly cultivated staple food grains, and ranks third in terms of global production, owing to about 35% of the world’s food grain produce (Obembe et al., 2021). It contributes significantly to the daily nutrient intake of the human population, thus providing plant-derived proteins, carbohydrates, calories, vitamins, and a wide range of other nutrients (Shewry and Hey, 2015; Miransari and Smith, 2019). The gradual increase in the world’s human population leads to increased threat to global food security, which further demands to improve the crop yield substantially to meet the food supply of the world in the future (El Sabagh et al., 2021). Apart from the climate change, the global wheat production is also affected by various unpredictable biotic and abiotic stresses, which further leads to reduced genetic diversity of the crop (Afzal et al., 2015). Annually, the diseases caused by plant-pathogenic fungi lead to yield losses varying from 15 to 20%. Among these pathogenic fungi, the obligately biotrophic rust fungi emerge as a major threat to wheat production, leading to an economic loss of $4.3–5.0 billion dollars (Figueroa et al., 2018).

Stem (black) rust, caused by *Puccinia graminis* f. sp. *tritici* (*Pgt*), is considered as one of the highly destructive diseases of wheat. The occurrences of the disease have also been found in crops such as barley, rye, and other cereals (Dean et al., 2012). The disease can cause enormous yield losses ranging from 50 to 70% or more, depending on the environmental conditions (Saari and Prescott, 1985). Stem rust is also responsible for contraction of grain size, reduced photosynthetic area, diversion of photosynthetic assimilates, and water loss (Willocquet et al., 2021). *Pgt* consists of a wide range of strains, the most significant being the African strain “Ug99” (race TTKSK), which later evolved into variants of its own (Olivera et al., 2012; Li et al., 2019). Another *Pgt* isolate, “21-0,” was found in Australia, which has been used for the comparative study of stem rust in wheat. The draft genome of *Pgt* 21-0 was built using reference-based and *de novo* assembly (Upadhyaya et al., 2015). The infection by *Pgt* occurs in a series of steps, typically initiating by the germination of urediniospores on the surface of plant stem, followed by the formation of appressorium, mitosis of nuclei, and differentiation of haustorial mother cells into haustoria, which acquires nutrients from the plant cells (Leonard and Szabo, 2005).

The tremendous losses caused by the fungal pathogens have spurred the researchers to study the in-depth infection mechanism of the pathogen. Various studies have progressed the detection and genetic mapping of genes, and QTLs that confer resistance to *Pgt* in wheat (Duplessis et al., 2011), but a frequent resistance breakdown has been observed, owing to mutations in the *Pgt* isolates (Stokstad, 2007). The fungicides are an effective way against the fungal pathogens, but these pathogens develop resistance against the fungicides/chemicals, and also the fungicides have a negative impact on human health and environment (Van de Wouw et al., 2014). The protein-protein interactions (PPIs) in plant cells perform various functions, involving immune responses against biotic or abiotic stresses. The pathogens secrete effector proteins into the plant cell, sabotage the intercellular mechanisms of the host cell, and cause infection (Garbutt et al., 2014). Thus, the understanding of pathogen infection and the subsequent plant cell defense response is highly crucial. Computational prediction of PPIs reveals relationship among the proteins on a genome-wide scale. Various computational methods exist for the prediction of host-pathogen interactions (HPIs) such as protein sequence homology-based interolog approach, domain-based approach, gene co-expression, phylogenetic profiles, and others (Matthews et al., 2001; Ng et al., 2003; Sun et al., 2007; Piya et al., 2014; Kataria et al., 2022). In the present study, we delineated the PPIs between *T. aestivum* and *Puccinia* species proteins by employing two most widely used computational approaches, i.e., interolog (homology-based) and domain-based approach. Different molecular strategies for PPI detection are available, but those are expensive, time-consuming, and labor-intensive (Chen et al., 2008). Our research is mainly focused on elucidating genome-wide scale PPIs to unravel the complex intermolecular networks of *T. aestivum*-*Puccinia* interactome.

## Materials and Methods

### Data Source

The whole proteomes of *T. aestivum*, *Pgt* isolate 21-0, and *Pgt* isolate Ug99 were obtained from Ensembl Plants,^1^ National Center for Biotechnology Information (NCBI),^2^ and Ensembl Fungi,^3^ respectively. All the proteomes were analyzed with CD-HIT (Fu et al., 2012) at 100% to cluster the identical proteins. The total number of proteins are described in Table 1. In the research analysis, the proteins with prefixes “Traes,” “KAA,” and “GMQ” refer to *T. aestivum*, *Pgt* 21-0, and *Pgt* Ug99 proteins, respectively.

**TABLE 1:** Protein datasets used in the study.

Species	Number of proteins	
	Downloaded	Non-redundant
*Triticum aestivum*	133,346	104,701
*Puccinia graminis* 21-0 (*Pgt* 21-0)	37,843	35,376
*Puccinia graminis* Ug99 (*Pgt* Ug99)	24,524	22,524

### Interactome Prediction Between *Triticum aestivum* and *Puccinia* Species

The HPIs between *T. aestivum* and *Puccinia* species were predicted using two most widely implemented computational approaches: interolog-based, and domain-based. Interolog method is based on sequence homology that determines the conserved interactions between protein pairs of two species (Nourani et al., 2015). The interolog-based approach employs seven protein-protein interaction (PPI) databases, viz., BioGRID (Chatr-Aryamontri et al., 2017), DIP (Salwinski et al., 2004), HPIDB (Kumar and Nanduri, 2010), IntAct (Kerrien et al., 2012), MINT (Licata et al., 2012), PHI-base (Urban et al., 2020), and STRING (Szklarczyk et al., 2019). The interaction data from these databases was downloaded and implemented locally using SQL. The proteomes of host and pathogen species are aligned against these PPI databases using BLAST v2.7.1, followed by filtering of the results using random BLAST parameter combinations of sequence identity (30, 40, 50, and 60%), sequence coverage (40, 50, 60, and 80%), and *e*-value (1*e*-10, 1*e*-50, 1*e*-05, 1*e*-04, 1*e*-20, 1*e*-30, and 1*e*-25). In the past, there are no substantial reports for selecting an appropriate combination of BLAST parameters to predict PPIs https://academic.oup.com/bib/article/22/3/bbz162/5842243. A study on human and *Escherichia coli* HPIs determined the homologs using 30% sequence identity, 80% coverage, and *e*-value ≤1*e*-10 (Bose et al., 2017). In Arabidopsis-*Pseudomonas* system, the researchers identified homologs with 80% coverage, 1*e*-04 *e*-value, and 50% identity (Sahu et al., 2014). In our study, using different BLAST parameters (identity, coverage, and *e*-value), 112 combinations were generated, and an optimal combination was selected based on the maximum number of effectors.

On the other hand, in the domain-based approach, three domain-domain interaction (DDI) databases were implemented locally: 3did (Mosca et al., 2014), DOMINE (Raghavachari et al., 2008), and IDDI (Kim et al., 2012). In this method, the PPIs are predicted on the basis of Pfam domain composition. The proteins of host and pathogen were analyzed against Pfam database using “hmmscan” program in HMMER v3.3.1, which identified the significant domains. To filter the results of hmmscan, an *e*-value of 1*e*-23 and coverage 0.2 was used for host proteins, while those of pathogen proteins were filtered with *e*-value and coverage of 1*e*-17 and 0.45, respectively. The identified Pfam domains were then further used for the prediction of PPIs using local SQL queries. The details of number of sequences, and interaction pairs from each database are available in Supplementary Material 1, Sheet 1.

### Effector and Secretory Proteins Prediction

Effector proteins, secreted by the fungi, interact with host proteins and manipulate the immune responses of host cell (Sonah et al., 2016). The secretory proteins contain a secretion signal peptide, less than 300 amino acids, that employs various cell wall degrading enzymes and phytotoxins to modulate the crucial host cell defense mechanisms (Kim et al., 2016). To identify the effector proteins, we analyzed the proteomes of *Pgt* 21-0 and *Pgt* Ug99 in EffectorP 2.0^4^ (Sperschneider et al., 2018), while the secretory proteins were identified using SignalP-5.0^5^ (Almagro Armenteros et al., 2019).

### Functional Enrichment Analysis of the Proteins

The classification of the proteins into different functional categories such as molecular function, biological process, and cellular component was carried out by obtaining the functional annotation of the proteins. Gene Ontology (GO) and Kyoto Encyclopedia of Genes and Genomes (KEGG) analyses were conducted using the clusterProfiler (Yu et al., 2012) package in R. GO databases for *T. aestivum* and *Puccinia* species was created locally using *makeOrgPackage* function of the R package “AnnotationForge.” GO enrichment was then performed by implying Benjamini and Hochberg test correction method (Benjamini et al., 1995), followed by filtering the enriched terms on adjusted *p*-value cutoff of ≤0.05. Similarly, KEGG enrichment was also conducted at a *p*-value cutoff of 0.05.

### Subcellular Localization

According to the studies, a high correlation is observed between the protein function and its subcellular localization, which provide more insights into the protein function (Chi, 2010). The pathogens secrete effector proteins into the host cell, which then translocate to various cellular compartments, and suppress the immune system of the host (Sperschneider et al., 2017). Thus, the prediction of subcellular localization of the host and pathogen proteins is an essential component of the plant-pathogen interaction studies. The subcellular localization of *T. aestivum* proteins was performed using standalone version of Support Vector Machine (SVM)-based tool, Plant-mSubP (Sahu et al., 2021). While for the subcellular localization of *Puccinia* proteins, we employed a deep learning-based tool, DeepLoc 1.0 (Almagro Armenteros et al., 2017).

### Comparison Between Host-Pathogen Interactions of *Pgt* 21-0 and *Pgt* Ug99

Different races of *P. graminis* cause stem rust infection in wheat. We were interested in comparing the PPIs between two major strains (*Pgt* 21-0 and *Pgt* Ug99) to uncover the differences between the two fungal species. With regard to this, we identified the orthologs between both the fungal species using OrthoFinder, which implements phylogenetic-based prediction of the orthologs (Emms and Kelly, 2019). The interactions from the orthologs were referred to as common subnetwork. Further, we also focused on the strain-specific functional analysis of the *Puccinia* species. For this, we analyzed the “unique proteins,” i.e., the *Puccinia* species proteins that were not the orthologs of each other. This provided us more insights into the functionality of an individual strain.

### Network Visualization and Analysis

The protein-protein interaction network is an extensively employed tool to study the functioning of cellular machinery by highlighting the crucial protein complexes and the relationship between the proteins, based on various network parameters such as node degree, centrality, etc. (Agapito et al., 2013). We analyzed the protein networks using the most widely used tool, Cytoscape v3.8.2 (Shannon et al., 1971). Various in-built layout algorithms and styles were used to analyze and enhance the visualization of the network.

## Results and Discussion

To predict the protein-protein interactions, the proteomes of *T. aestivum* and *Puccinia* species were randomly paired, followed by the estimation of the interaction probability of an individual pair using interolog, and domain-based approaches. The interactome was predicted using the BLAST parameter combination of 30% sequence identity, 40% sequence coverage, and *e*-value of 1*e*-04. Using both the computational approaches, the predicted interactome for *T.* aestivum-*Pgt* 21-0 consisted of 90,493,282 interactions, whereby 84,125 host proteins interact with 9,022 pathogen proteins, of which 115 proteins were effectors (Table 2). While the *T. aestivum*-*Pgt* Ug99 interactome accounted for 56,755,414 interactions, involving 84,069 host and 5,863 pathogen proteins, consisting of 34 effectors (Table 3). The randomly employed (112) BLAST parameter combinations, and the resulting interactions from each combination for *Pgt* 21-0 and *Pgt* Ug99 have been described in Supplementary Material 1, Sheets 2, 3, respectively. For clarification, the term “effectors” has been used to represent the pathogen proteins that serve both as effector and secretory proteins.

**TABLE 2:** *Triticum aestivum*-*Pgt* 21-0 interactome.

Interaction database	Number of interactions	Number of host proteins	Number of pathogen proteins
**Interolog-based**			
BioGRID	22,129,912	53,400	7,214
DIP	2,484,487	27,287	4,839
HPIDB	62,505	6,844	418
IntAct	8,870,790	49,486	6,641
MINT	2,497,106	23,672	5,251
PHI-base	154	7	22
STRING	60,582,077	83,058	5,369
Total (Interolog) (I)	73,877,190	83,821	7,758
**Domain-based**			
3DID	2,336,648	27,053	4,891
DOMINE	11,148,777	25,649	5,130
IDDI	22,963,441	33,862	5,982
Total (Domain) (II)	27,163,377	35,734	6,305
I and II (combined)	90,493,282	84,125	9,022
I and II (consensus)	10,547,285	31,143	4,689
Interolog (unique)	63,329,905	83,816	7,755
Domain (unique)	16,616,092	34,159	6,190

*Total (Interolog) (I): The predicted HPIs from all the seven interolog databases were merged and duplicates were removed.*

*Total (Domain) (II): The predicted HPIs from all the three domain databases were merged and duplicates were removed.*

*I and II (combined): The predicted HPIs from both the methods were merged and the duplicates were removed.*

*I and II (consensus): From both the methods, the consensus of the predicted HPIs was taken and duplicates were removed.*

*Interolog (unique): The unique HPIs containing the interactions only from interolog-based method.*

*Domain (unique): The unique HPIs containing the interactions only from domain-based method.*

**TABLE 3:** *Triticum aestivum*-*Pgt U*g99 interactome.

Interaction database	Number of interactions	Number of host proteins	Number of pathogen proteins
**Interolog-based**			
BioGRID	14,058,763	53,443	4,453
DIP	1,613,309	27,005	3,021
HPIDB	41,663	6,883	266
IntAct	5,752,184	48,653	4,122
MINT	1,641,961	23,550	3,286
PHI-base	77	7	11
STRING	38,432,627	83,007	3,620
Total (Interolog) (I)	46,736,430	83,767	5,104
**Domain-based**			
3DID	1,510,939	27,010	2,942
DOMINE	6,770,500	25,471	3,117
IDDI	13,827,014	33,824	3,602
Total (Domain) (II)	16,528,057	35,737	3,809
I and II (combined)	56,755,414	84,069	5,863
I and II (consensus)	6,509,073	30,901	2,834
Interolog (unique)	40,227,357	83,759	5,100
Domain (unique)	10,018,984	34,272	3,741

*Total (Interolog) (I): The predicted HPIs from all the seven interolog databases were merged and duplicates were removed.*

*Total (Domain) (II): The predicted HPIs from all the three domain databases were merged and duplicates were removed.*

*I and II (combined): The predicted HPIs from both the methods were merged and the duplicates were removed.*

*I and II (consensus): From both the methods, the consensus of the predicted HPIs was taken and duplicates were removed.*

*Interolog (unique): The unique HPIs containing the interactions only from interolog-based method.*

*Domain (unique): The unique HPIs containing the interactions only from domain-based method.*

### *Puccinia* Orthologs Interactome

The ortholog analysis resulted in 1,958 proteins that are orthologs between *Pgt* 21-0 and *Pgt* Ug99. These orthologs were found to interact with 83,340 host proteins, involved in 21,901,125 interactions (referred to as “common subnetwork” throughout the analysis). For the subsequent functional analysis, the interactions from ortholog analysis were taken into consideration.

#### Highly Connected Protein Hubs

The host-pathogen protein-protein interaction network represents the functional clustering of the interacting proteins, which allows in-depth understanding of a specific protein with respect to the proteins in its surrounding (Wachi et al., 2005; Jonsson and Bates, 2006). The identification of the protein function helps gaining the knowledge of the disease infection mechanism by providing information about various biological processes and molecular mechanisms (Kuzmanov and Emili, 2013). In our study, the proteins hubs were determined from common subnetwork using the metric “node degree.” The average degree of host and pathogen proteins was found to be 263 and 11,185, respectively (Supplementary Material 2, Sheets 1, 2). The pathogen proteins have higher degree in comparison to host proteins, which is in-line with the host-pathogen protein ratio obtained by the researchers in the past (Li et al., 2012; Kurubanjerdjit et al., 2013). The top 20 protein hubs for each host and pathogen have been discussed below.

##### *Triticum aestivum* Protein Hubs

**FIGURE 1:**
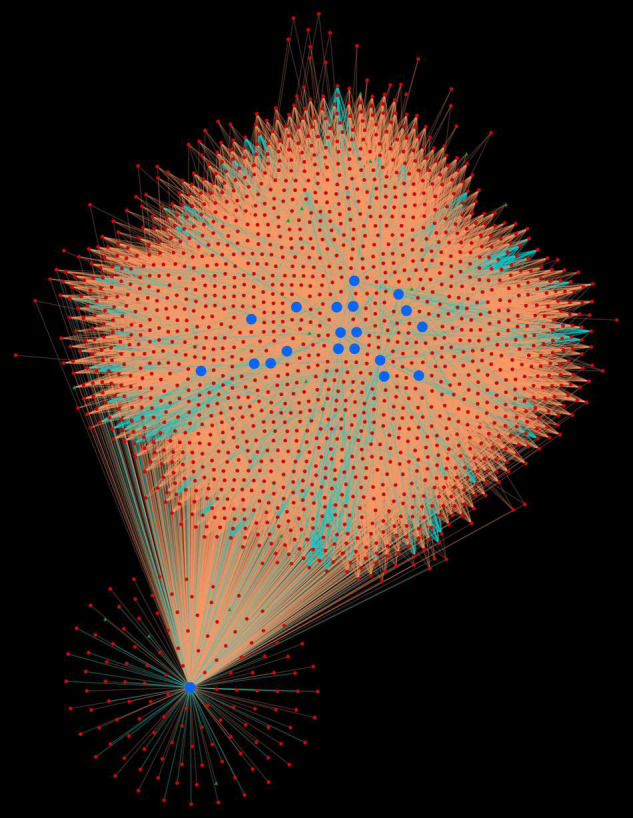
Protein-protein interaction network for top 20 host protein hubs. Blue nodes represent host proteins, red nodes are pathogen proteins, and green nodes are effector proteins. Orange edges depict the interactions from interolog-based approach, while cyan edges belong to domain-based approach.

The protein network analysis revealed that majority of the host proteins belonged to protein kinase domain family, of which serine/threonine (Ser/Thr) protein kinase was found to form highly interconnected hubs (TraesCS4D02G250600.1.cds1, TraesCS7A02G437400.1, TraesCS5B02G254600.1.cds1, TraesCS 5A02G255500.1.cds1, TraesCS5D02G263800.1.cds1, TraesCS 2D02G120200.1, TraesCS3A02G247700.1.cds1, TraesCS4B02G2 60700.1.cds1, TraesCS3B02G271700.1.cds1, TraesCS4B02G1231 00.1, and TraesCS4A02G192300.1) (Figure 1). The physiological importance of Ser/Thr kinases play a crucial role in the regulation of various environmental stress responses, particularly in signaling pathway (País et al., 2009). SnRK Ser/Thr kinase is divided into 3 subgroups: SnRK1, SnRK2, and SnRK3 (Mao et al., 2010). In wheat, PKABA1 (a member of SnRK2) has been reported to be induced by ABA. The levels of ABA increase during stress conditions, thus showing its role in plant defense (Johnson et al., 2002). Another most interconnected protein (TraesCS6D02G339600.1), interacting with 1,497 pathogen proteins, belonged to heat shock protein 70 (hsp70) family. The members of hsp70 are thought to play a crucial role in different cellular processes during biotic and abiotic stress conditions (Usman et al., 2017). These proteins are also involved in R protein stability, and regulation of immune signaling pathways (Van Ooijen et al., 2010; Park and Seo, 2015). Five host proteins (TraesCS5A02G295800.1, TraesCS7A02G029700.1, TraesCS4A02G336800.2, TraesCS5B02G536500.1, and TraesCS5D02G534000.2) were identified as mitogen-activated protein kinases (MAPKs), which are known to be critical in response to pathogenic infection including the generation of hypersensitive response (HR), defense hormone responses, and ROS signaling (He et al., 2020). Another major hub was formed by the proteins (TraesCS5A02G521700.1, TraesCS4B02G353600.1, and TraesCS4D02G347600.1) that function as cyclin-dependent kinases (CDKs). A study shows the regulation of Arabidopsis resistance against *Alternaria brassicicola* by CDK8 that regulates the intermediates of the secondary metabolites, hydroxycinnamic acid amides (HCAAs), that play a role in fungal resistance. Also, an increased resistance against *Botrytis cinerea* was observed in the *cdk8* mutant (Bessire et al., 2007; Zhu et al., 2014).

##### *Puccinia* Protein Hubs

The common subnetwork analysis indicated that the largest hub was formed by aurora kinase that involves the *Puccinia* species protein GMQ_15838T0/KAA1117900.1, interacting with 57,744 host proteins. Various studies reveal the role of aurora kinase in mitotic processes such as chromosome segregation and cytokinesis, thus promoting the growth of fungal pathogens (Tückmantel et al., 2011; Bavetsias and Linardopoulos, 2015). Heat shock protein 70 superfamily forms another major hub involving seven pathogen proteins GMQ_09878T0/KAA1086976.1, GMQ_23673T0/KAA1112990.1, GMQ_10843T0/KAA107691 6.1, GMQ_14817T0/KAA1118905.1, GMQ_14422T0/KAA108 6735.1, GMQ_13441T0/KAA1119794.1, and GMQ_05646 T0/KAA1072403.1. Heat shock proteins are conserved molecular chaperones that play characteristic role in activating essential signal transducers in pathogenic fungi (Tiwari et al., 2015). In *Fusarium pseudograminearum*, 14 *FpHsp70* genes were highly expressed at the time of crown rot infection in wheat. While the knockout of a Hsp70 homolog gene (*FpLhs1*) in ER lumenal resulted in reduction of fungal growth and virulence, implying the role of HSPs in pathogenicity (Chen et al., 2019). The pathogen proteins (GMQ_03421T0/KAA1111489.1, GMQ_12489T0/KAA1117418.1, GMQ_08991T0/KAA108750 2.1, GMQ_13869T0/KAA1085281.1, and GMQ_15253T0/KAA1 066088.1) were found to function as calcium/calmodulin-dependent kinases (CaMKs). Based on hidden Markov model, the fungal CaMKs are classified into different families (CAMK1, CAMKL, RAD53, and CAKM-Unique), and subfamilies (Kin4, Kin1, GIN4, PASK, AMPK, CHK1, and MARK) (Goldberg et al., 2013; Jiao et al., 2017). *Fg*Kin1 and *Fg*Kin4 in wheat fungal pathogen, *Fusarium graminearum*, are reported to be responsible for growth and pathogenesis (Wang et al., 2011; Luo et al., 2014). Similarly, the pathogen hubs formed by two Ser/Thr protein kinases: glycogen synthase kinase (GMQ_16722T0/KAA1117762.1), and AGC kinase (GMQ_11492T0/KAA1086570.1) are critical for pathogenicity and development in fungi (Qin et al., 2015; Fabri et al., 2019). The cluster of proteins (GMQ_05648T2/KAA1078421.1, GMQ_24430T0/KAA1114232.1, and GMQ_09263T0/KAA11 11598.1) served as cyclin-dependent kinases (CDKs). Researchers in the past established that the rice blast fungus, *Magnaporthe oryzae*, requires CDK subunit Cks1 for infection-associated development (Yue et al., 2017). Two proteins, GMQ_11353T0/KAA1076537.1 and GMQ_19044T0/KAA1114913.1, were associated with RNA-binding domain/RNA recognition motif. In *Ustilago maydis*, the causal agent of smut disease in corn, RNA-binding proteins were found to be involved in the fungal growth and development during the infection process (Becht et al., 2005).

The protein hubs analysis indicated that the pathogen proteins invade and subvert the host immune machinery, while the host activates various signaling cascades and hormones in response to the pathogen attack. Additionally, the hubs significantly revealed the crucial protein domain families that are involved in the disease infection and defense mechanisms in the pathogen and host, respectively, thus suggesting the cross-talks between the host and pathogen.

#### Gene Ontology Analysis: Unifying the Biology of Host and Pathogen Proteins

**FIGURE 2:**
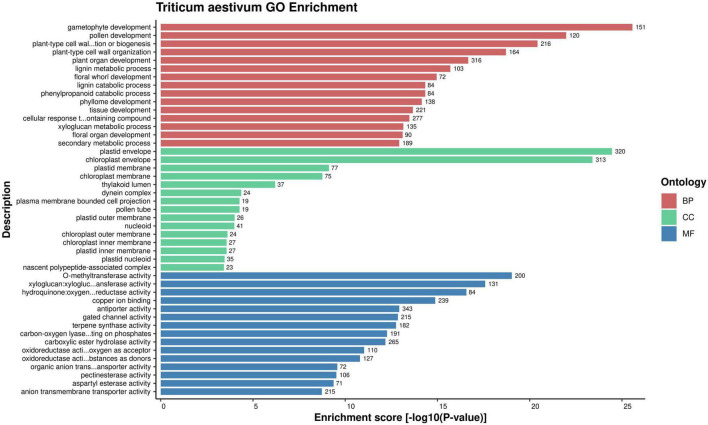
Over-representation of top 15 GO terms from each category (molecular function, cellular component, and biological process) for the host proteins, based on enrichment score.

**FIGURE 3:**
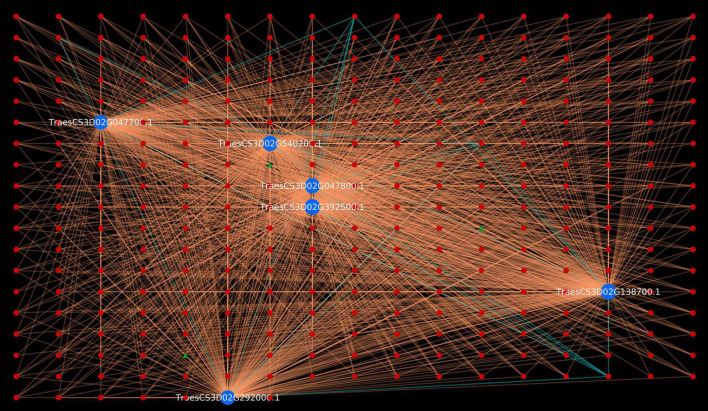
Visualization of six *Triticum aestivum* proteins belonging to chromosome 3D in GO:0008171 (*O*-methyltransferase activity). Blue nodes represent host proteins, red nodes are pathogen proteins, and green nodes are effector proteins. Orange edges depict the interactions from interolog-based approach, while cyan edges belong to domain-based approach.

Gene Ontology enrichment analysis is an effective approach of deciphering the underlying biological process, molecular function and cellular component of the proteins of an organism (Tomczak et al., 2018). GO enrichment of the host and pathogen proteins was carried out using enrichment score [-log10(*P*-value)]. The enrichment analysis revealed that 83,340 host proteins in the common subnetwork are involved in 3,570 GO terms, categorized into biological process (2,167), cellular component (408), and molecular function (995) (Figure 2). The highly enriched GO terms in different categories involve gametophyte development (GO:0048229), regulation of response to stimulus (GO:0048583), plastid envelope (GO:0009526), chloroplast envelope (GO:0009941), *O*-methyltransferase activity (GO:0008171), xyloglucan:xyloglucosyl transferase activity (GO:0016762), and other significant GO terms (Supplementary Material 2, Sheet 3). Various studies have reported the direct or indirect involvement of the above-mentioned significant biological processes/cellular components/molecular functions in plant defense mechanisms. Ubiquitin-conjugating enzymes (E1, E2, and E3) are associated with ubiquitination, which regulates various plant immune signals. In Arabidopsis, UBC22 (E2 subfamily) showed its involvement in female gametophyte development, indicating its role in plant defense (Devoto et al., 2003; Wang et al., 2016). The enzyme hydroperoxide lyase (HPL) in plastid envelope is known to catalyze C6-aldehyde that play a role in plant defense. The attack of pathogenic fungi, *B. cinerea*, on Arabidopsis showed an upregulation of AtHPL expression, thus enhancing C6-aldehyde levels, which further inhibited the pathogen growth (Howe and Schilmiller, 2002; Kishimoto et al., 2008; Breuers et al., 2011). Caffeic acid 3-*O*-methyltransferases (COMT) are implicated in biosynthesis of lignin, which provides biotic/abiotic stress resistance to the plants (Bhuiyan et al., 2009; Kataria and Kaundal, 2021). In wheat, the COMT gene (*TaCOMT-3D*) showed significantly high expression level on infection with *Rhizoctonia cerealis*. *TaCOMT-3D* was localized in chromosome 3D (Wang et al., 2018). In our analysis, we identified six wheat proteins (TraesCS3D02G392500.1, TraesCS3D02G540200.1, TraesCS3D02G047700.1, 394TraesCS3D02G047800.1, TraesCS3D02G138700.1, and TraesCS3D02G292000.1) that belong to chromosome 3D and are associated with *O*-methyltransferase activity. These six host proteins were found interacting with 307 pathogen proteins, accounting to 1,611 interactions (Figure 3). This provides concrete evidence of the involvement of host proteins in plant defense against fungal attack. Furthermore, lignification also restricts the diffusion of nutrients from host to pathogen, thus suggesting the inability of haustoria to maintain the biotrophic relationship with the host, resulting in reduced pathogen infection.

On the other hand, 1958 pathogen proteins associated with the host proteins were involved in 1,362 GO terms. These include significant GO terms such as proteolysis (GO:0006508), protein peptidyl-prolyl isomerization (GO:0000413), endoplasmic reticulum (GO:0005783), GTPase activity (GO:0003924), and hydrolase activity (GO:0004553). These processes are known to be involved in pathogen virulence and development (Pogány et al., 2015; Pinter et al., 2019). The detailed GO enrichment of *Puccinia* species has been provided in Supplementary Material 2, Sheet 4.

#### Plant Defense and Immune Signaling Pathways During Biotic Stress

**FIGURE 4:**
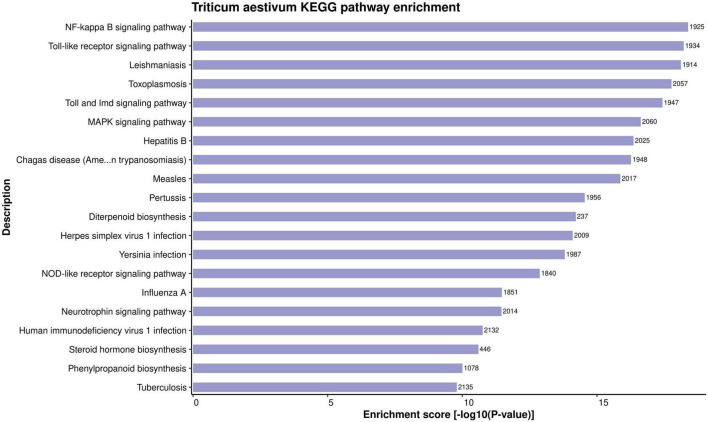
Representation of top 20 enriched KEGG pathways for the host proteins involved in HPIs, based on enrichment score.

The in-depth knowledge of biological pathways of the proteins helps in better understanding of a PPI network. KEGG enrichment analysis of the proteins involved in PPIs was conducted. A total of 399 highly enriched KEGG pathways were obtained for the host proteins in the common subnetwork (Supplementary Material 2, Sheet 5). The over-represented pathways include NF-kappa B signaling pathway (ko04064), flavonoid biosynthesis (ko00941), biosynthesis of secondary metabolites (ko01110), plant-pathogen interaction (ko04626), MAPK signaling pathway (ko04016), and a few more significantly enriched pathways related to plant defense mechanism. The top 20 KEGG pathways have been represented in Figure 4. The nuclear factor kappa B (NF-κB) transcription factor helps in the regulation of cellular immune responses against diverse environmental stresses (Zhao et al., 2018). The interaction of protein NIM1 with transcription factor NF-κB has been reported to induce systemic acquired resistance (SAR) and gene-for-gene resistance against the disease in Arabidopsis (Ryals et al., 1997). The secondary metabolites are known to play a major role in plant immune responses to external stimuli. In our analysis, around 4,640 host proteins were found to be involved in biosynthesis of secondary metabolites pathway. Previous reports show the activation of secondary metabolites on recognition of the pathogen-secreted effectors by resistance proteins in the host (Ahuja et al., 2012; Piasecka et al., 2015). Flavonoids, a class of secondary metabolites, have been reported to account for plant development and defense responses against pathogens in various crops such as cotton (Mathesius, 2018). Among the aforementioned pathways, the most significant is mitogen-activated protein kinase (MAPK) signaling pathway, which is known to play a critical role in plant immune signaling during various stresses (Zhang and Klessig, 2001). A study revealed the induction of rice MAPKs, OsMKK3, and OsMPK7, during the infection process of *Xanthomonas oryzae* that causes leaf blight disease in rice. The overexpression of OsMKK3 and OsMPK7 genes during pathogenesis also suggested the probable disease resistance mechanism. Also, the silencing of overexpressed OsMPK7 lead to disease susceptible plants (Jalmi and Sinha, 2016). Also, transcription factors are known to be activated by MAPKs by the process of phosphorylation, thus regulating the immune response against the pathogens by integrating defense signals from various MAPKs (Nadal-Ribelles et al., 2019).

We also performed the KEGG pathway enrichment of the pathogen proteins, which revealed important pathways such as MAPK signaling pathway (ko04016), biosynthesis of secondary metabolites (ko01110), and others, which have a direct or indirect relationship with pathogenicity (Supplementary Material 2, Sheet 6). The host proteins are also involved in these pathways, which suggests the potential interaction of host and pathogen proteins during plant defense response.

Thus, the functional enrichment analysis suggested significant molecular processes and biological pathways in which the host and pathogen proteins are involved. A comprehensive analysis of the enriched processes/pathways can further enhance the study of host-pathogen interaction mechanism, and other related biological processes occurring within the host cell.

#### Plastid: A Primary Target for Pathogen Attack in the Host

The proteins are translocated to various subcellular compartments, where they perform specific biological functions, thus revealing the physiology of the cell. Some proteins are also distributed to multiple cellular locations, depending on the sorting signal (Briesemeister et al., 2010). The subcellular localization is also known to be statistically correlated with the protein function, and its gene expression levels (Garapati et al., 2020). A significant number of novel proteins have been identified using sequencing technologies, but their subcellular location remains unknown.

**FIGURE 5:**
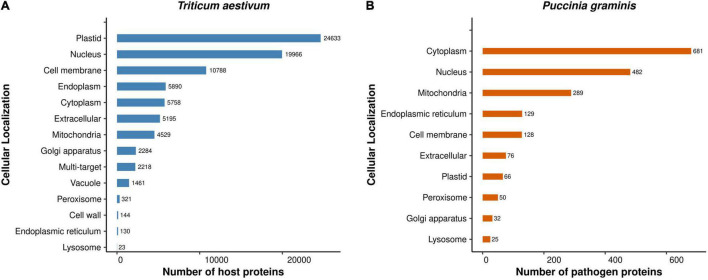
Subcellular Localization of the **(A)** host proteins, and **(B)** pathogen proteins involved in the common subnetwork.

We predicted the sequence-based subcellular localization of the *T. aestivum* and *Puccinia* proteins to have a better understanding of the occurrence of PPIs in a particular subcellular compartment. The subcellular localization analysis classified the *T. aestivum* proteins into 14 categories: plastid (29.56%), nucleus (23.96%), cell membrane (12.94%), endoplasm (7.07%), cytoplasm (6.91%), extracellular (6.23%), mitochondria (5.43%), golgi apparatus (2.74%), multi-target (2.66%), vacuole (1.75%), peroxisome (0.38%), cell wall (0.17%), endoplasmic reticulum (0.16%), and lysosome (0.03%) (Figure 5A). 2,218 proteins were found to be multi-target (moonlighting proteins), performing specific functions in various cellular organelles. 24,633 and 19,966 host proteins were localized in plastid and nucleus, respectively. Researchers in the past have reported the presence of host proteins in plastid, which plays an essential role in intracellular signaling pathways (de Dios Barajas-López et al., 2013; Caplan et al., 2015). Another study demonstrated the localization of rice OsVQ domain proteins in plastid and nucleus using rice protoplast system. OsVQ proteins are considered to be the co-regulators during immune response against biotic stress (Kim et al., 2013).

On the other hand, the *Puccinia* species proteins were localized in cytoplasm (34.78%), nucleus (24.62%), mitochondria (14.76%), endoplasmic reticulum (6.54%), cell membrane (6.54%), extracellular (3.88%), plastid (3.37%), peroxisome (2.55%), golgi apparatus (1.63%), and lysosome (1.28%) (Figure 5B). In *B. cinerea*, two ubiquitin-like (UBL) activating enzymes, BcAtg3 (E2) and BcAtg7 (E1), were determined to be localized in cytoplasm (Ren et al., 2018), which is coherent to our localization prediction. The detailed information of the predicted subcellular localization of the host and pathogen proteins is available in Supplementary Material 2, Sheets 7, 8, respectively. Furthermore, we were also interested in predicting the location of interactions between the host and pathogen proteins. The analysis indicated that the host proteins are mostly targeted by the pathogen proteins in the plastid, whereby 24,237 host proteins interact with 674 pathogen proteins, accounting to 2,630,496 interactions (Supplementary Material 2, Sheet 9).

#### Pathogenicity of Effectors During Stem Rust Infection

The rust fungal pathogens secrete effectors (using specialized structures known as haustoria) which subvert the host cell immune machinery, followed by enhancing their pathogenicity in the host cell (Ramachandran et al., 2017). Therefore, understanding the behavior of the effector proteins is a crucial step in the HPI analysis. From the common subnetwork, we identified 18 effectors interacting with 43,054 host proteins, resulting in 156,529 interactions (Supplementary Material 3, Sheet 1). Among the 43,054 host proteins involved in interactions with effectors, 3367 were identified to be transcription factors.

The functional analysis suggested that the effector proteins in the interactions are highly enriched in superoxide metabolic process (GO:0006801), protein dephosphorylation (GO:0006470), vesicle (GO:0031982), phosphoric ester hydrolase activity (GO:0042578), and metabolic pathways (ko01100). These processes are involved in enhancing pathogenicity, development, and secretion during the host-pathogen interaction mechanism (Rodrigues et al., 2011; Tamayo et al., 2016; Rafiei et al., 2021), which helps in the survival of the pathogen under various stresses in the host cell. The host proteins associated with the effectors were found to be involved in secondary metabolic process (GO:0019748), cell wall polysaccharide metabolic process (GO:0010383), response to external biotic stimulus (GO:0043207), plant-pathogen interaction pathway (ko04626), and plant hormone signal transduction (ko04075). These processes are actively related to the plant defense and immune signaling against the biotic stresses. Thus, the predicted interactions of host proteins with the effectors can be considered of high confidence, and potential candidates for further studying the infection mechanism of stem rust in wheat.

### Functional Differences Between *Pgt* 21-0 and *Pgt* Ug99

Furthermore, we were interested in deciphering the strain-specific functionalities of the *Pgt* 21-0 and *Pgt* Ug99 proteins involved in the PPIs. Since these proteins were not the orthologs of each other, hence these are referred to as unique proteins. The *T. aestivum*-*Pgt* 21-0 interactome predicted 68,465,557 PPIs, involving 83,962 host proteins and 7,063 unique *Pgt* 21-0 proteins, of which 100 proteins served as effectors. While the *T. aestivum*-*Pgt* Ug99 interactome involved 83,495 host proteins and 3,905 *Pgt* Ug99 proteins (16 effectors), accounting to 34,854,274 interactions. The low number of pathogens and predicted PPIs in *Pgt* Ug99 interactome as compared to that of *Pgt* 21-0 suggests the virulence of the pathogen, and that fewer pathogen proteins (mainly effectors) are capable of causing the infection.

#### Unique *Puccinia graminis* 21-0

The GO enrichment analysis of the unique pathogen proteins suggested that most of the GO terms were similar to that of the common subnetwork. But we also found 180 significant GO terms that were unique to *Pgt* 21-0 proteins. These included cell wall modification (GO:0042545), NADH dehydrogenase complex assembly (GO:0010257), cyclin-dependent protein kinase holoenzyme complex (GO:0000307), and SUMO ligase complex (GO:0106068). The unique KEGG pathways such as carotenoid biosynthesis (ko00906), and plant hormone signal transduction (ko4075) were found to be highly over-represented. Researchers in past have reported the direct or indirect role of these GO terms/KEGG pathways in pathogen virulence (Avalos et al., 2017). The detailed information of the significant GO terms and KEGG pathways for *Pgt* 21-0 is available in Supplementary Material 3, Sheets 2, 3, respectively. For the host proteins interacting with the unique *Pgt* 21-0 proteins, only 1 significant GO term (condensed chromosome kinetochore; GO:0000777) was identified. While no unique KEGG pathway was obtained for the host proteins.

#### Unique *Puccinia graminis* Ug99

In comparison with the common subnetwork, the unique *Pgt* Ug99 were highly enriched in 201 GO terms, including oxidoreductase activity (GO:0016491), regulation of response to stress (GO:0080134), snoRNA binding (GO:0030515), and GTPase complex (GO:1905360). The over-represented KEGG pathways obtained for these proteins involve monoterpenoid biosynthesis (ko00902), and polyketide sugar unit biosynthesis (ko00523), which have been shown to regulate the fungal growth and development (Chiang et al., 2009; Dallery et al., 2019; Noar et al., 2019). The significant GO terms and over-represented KEGG pathways have been detailed in Supplementary Material 3, Sheets 4, 5, respectively. While no significant GO terms or KEGG pathways were found for the host associated with unique *Pgt* Ug99 proteins.

### Role of Transcription Factors in Plant Defense

Recent molecular studies have elucidated the role of transcription factors (TFs) in diverse cellular mechanisms such as regulating gene expression, act as transcriptional activators or repressors, and in plant defense (Seo and Choi, 2015). The plant immune signal activation is tightly controlled by the gene-specific transcription factors that bind to *cis*-elements in the promoter region (Li et al., 2016). In line with this, we predicted the wheat proteins that serve as transcription factors using PlantTFDB v5.0 (Jin et al., 2017). This resulted in 5,577 wheat proteins that served as transcription factors in the common subnetwork, involved in 1,311,301 interactions with 1,600 *Puccinia* ortholog proteins. These transcription factors were classified into 55 TF families, of which 28 families are significantly involved in biotic and abiotic stresses. The major TF families include basic helix-loop-helix (bHLH), ethylene responsive factor (ERF), myeloblastosis related (MYB), WRKY, basic leucine zipper (bZIP), and NAM, ATAF1/2, and CUC2 (NAM). The host proteins and their respective TF family has been described in Supplementary Material 4 (Sheet 1). Researchers in the past have extensively demonstrated the crucial role of various transcription factors in diverse biological processes, and significant immune signaling pathways in response to plant defense against pathogen attack (Asai et al., 2002; Wu et al., 2009; Zander et al., 2010; Pieterse et al., 2012; Zhao et al., 2012).

### Novel Stem Rust-Resistant Host Targets

Our study on the host-pathogen interaction system focuses on understanding the disease infection mechanism, host immune response, and identifying the host targets that show resistance against stem rust disease. The resistance (*R*) genes in host are responsible for the recognition of effector proteins (secreted by pathogens), followed by the initiation of immune responses. According to gene-for-gene hypothesis, a successful resistant response requires two genes: *R* gene in the host and corresponding avirulence (*Avr*) effector gene in the pathogen, which makes resistance dependent on the specific pathogen strain (Flor, 1971). The mutations in *Avr* genes leads to the inability of the corresponding *R* genes to recognize the *Avr* genes, thus resulting in the pathogen to overcome host resistance (Ellis et al., 2014). The recent advancement in plant breeding techniques, in conjunction with increasing genomic resources, has accelerated the identification (and cloning) process of wheat resistance genes (Andersen et al., 2020). Scientists also created a wheat *R*-gene atlas to facilitate the research community with an efficient resource of resistance genes in wheat, aiming at reducing the pathogen co-evolution (Hafeez et al., 2021).

In wheat, a total of 46 *R* genes are officially designated to show resistance against stem rust, of which only 20 belong to *T. aestivum* (Leonard and Szabo, 2005). A few of the identified *R* genes in wheat include *Sr5*, *Sr13*, *Sr23*, *Sr27*, *Sr36*, *Sr40*, etc., which have varying effect on *Pgt* races. In wheat, *R* gene *Sr5* is known to limit the growth of avirulent *Puccinia* strain, while *Sr22* advances the development of *Pgt* races (Hatta et al., 2020; Wu et al., 2020). Various researchers mapped *Sr* genes on wheat chromosomes 1BS, 2B, 3B, 5DL, 6AL, 6DS, and 7AL (Table 4), conferring resistance against stem rust during adult-plant stage. A QTL-based study on wheat identified stable QTLs: *QSr-sparc-2B*, *QSr-sparc-7A*, *QSr-sparc-5A*, *QSr-sparc-6A*, and *QSr-sparc-7B* on chromosome 2BS, 7AL, 5AL, 6AS, and 7BL, respectively (Bokore et al., 2021). Further, we identified the PPIs associated with these chromosomes, which accounted to 9,387,396 PPIs, involving 35,809 host and 1,956 pathogen proteins. Of the 1,956 pathogen proteins in the interactions, 18 proteins were identified as effectors, involved in 66,772 PPIs (Supplementary Material 4, Sheet 2). The maximum number of interactions (1,305,326 PPIs) were identified on chromosome 2B, on which five *Sr* genes (*Sr19*, *Sr23*, *Sr28*, *Sr36*, and *Sr40*) have been mapped. The plant immune response against various pathogens is mediated by nucleotide-binding and leucine-rich repeat (NLR) domain proteins. Among the identified 35,809 host proteins, 2,123 proteins were found to be associated with NLR domain.

**TABLE 4:** Stem rust resistance genes and QTLs mapped on various *T. aestivum* chromosomes.

	Gene/QTL	Chromosome number	References
Stem rust resistance genes	*Sr2*	3BS	Spielmeyer et al., 2003
	*Sr12*	3B	Crossa et al., 2007
	*Sr19*	2B	
	*Sr23*	2B	
	*Sr26*	6AL	
	*Sr31*	1BS	
	*Sr36*	2B	
	*Sr40*	2B	
	*Sr13*	6AL	Admassu et al., 2011
	*Sr22*	7AL	Upadhyaya et al., 2014
	*Sr28*	2BL	Rouse et al., 2012
	*Sr30*	5DL	Hiebert et al., 2010
	*Sr42*	6DS	Ghazvini et al., 2012
Stem rust resistance QTLs identified	*QSr-sparc-2B*	2BS	Bokore et al., 2021
	*QSr-sparc-7A*	7AL	
	*QSr-sparc-5A*	5AL	
	*QSr-sparc-6A*	6AS	
	*QSr-sparc-7B*	7BL	

**FIGURE 6:**
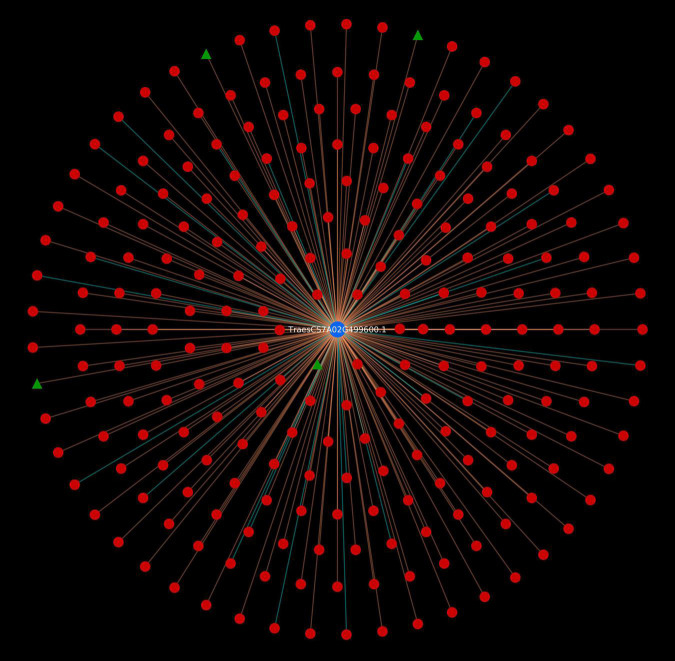
Wheat stem resistance gene *Sr22* encoded protein. Blue nodes represent host protein, red nodes are pathogen proteins, and green nodes are effector proteins. Orange edges depict the interactions from interolog-based approach, while cyan edges belong to domain-based approach.

**FIGURE 7:**
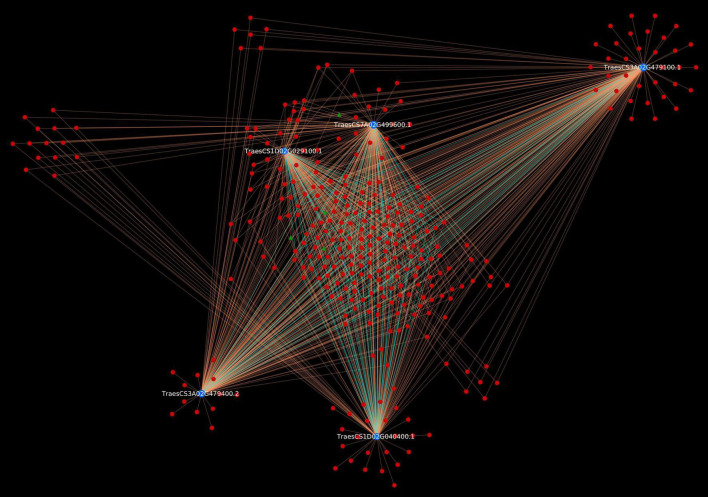
Stem rust resistance gene complex (*Sr22*, *Sr33*, *Sr35*, and *Sr45*) causing resistance against TTKSK race. Blue nodes represent host proteins, red nodes are pathogen proteins, and green nodes are effector proteins. Orange edges depict the interactions from interolog-based approach, while cyan edges belong to domain-based approach.

To have deeper insight into the resistance mechanism, we analyzed *Sr22* gene located on chromosome 7A and cloned using MutRenSeq (Steuernagel et al., 2016). In the predicted interactome, the protein encoded by this gene was found to be interacting with 230 pathogen proteins (230 PPIs). The host protein was actively involved in various plant defense pathways such as MAPK signaling, plant-pathogen interaction, and plant hormone signal transduction pathway. Additionally, 4 effectors were also identified (Figure 6). It has also been reported that the stem rust resistance genes (*Sr22*, *Sr33*, *Sr35*, and *Sr45*) form a complex that effectively confers resistance against TTKSK in wheat (Hatta et al., 2021). Further, we identified the interactions associated with this complex in the predicted interactome, which resulted in 1,051 PPIs, involving 327 pathogen proteins, of which 4 were effectors (Figure 7). The host proteins involved in the interactions can be considered as the novel targets for the breeders for development of disease-resistant lines. Further, the interaction of these novel host proteins with the effectors are the potential candidate pairs to understand the immune responses against the fungal pathogen attack during stem rust disease, thus giving deeper insights to the infection mechanism and host defense responses.

## Data Availability Statement

The original contributions presented in this study are included in the article/Supplementary Material, further inquiries can be directed to the corresponding author.

## Author Contributions

RKu formulated and designed the research and contributed to writing – review and editing, supervision, project administration, and funding acquisition. RKt analyzed the data, developed prediction models, performed functional analysis of the data, validations, literature mining, etc., and contributed to writing – original draft preparation. Both authors contributed to visualization, read, and agreed to the published version of the manuscript.

## Conflict of Interest

The authors declare that the research was conducted in the absence of any commercial or financial relationships that could be construed as a potential conflict of interest.

## Publisher’s Note

All claims expressed in this article are solely those of the authors and do not necessarily represent those of their affiliated organizations, or those of the publisher, the editors and the reviewers. Any product that may be evaluated in this article, or claim that may be made by its manufacturer, is not guaranteed or endorsed by the publisher.

## Footnotes

1
https://plants.ensembl.org/index.html

2
https://www.ncbi.nlm.nih.gov/

3
https://fungi.ensembl.org/index.html

4
http://effectorp.csiro.au/

5
https://services.healthtech.dtu.dk/service.php?SignalP-5.0
